# Immunoprofiling in Neuroendocrine Neoplasms Unveil Immunosuppressive Microenvironment

**DOI:** 10.3390/cancers12113448

**Published:** 2020-11-19

**Authors:** Antonia Busse, Liliana H. Mochmann, Christiane Spenke, Ruza Arsenic, Franziska Briest, Korinna Jöhrens, Hedwig Lammert, Bence Sipos, Anja A. Kühl, Ralph Wirtz, Marianne Pavel, Michael Hummel, Daniel Kaemmerer, Richard P. Baum, Patricia Grabowski

**Affiliations:** 1 Department of Hematology, Oncology and Tumor Immunology, Campus Benjamin Franklin, Charité–Universitätsmedizin Berlin, Corporate Member of Freie Universität Berlin, Humboldt-Universität zu Berlin and Berlin Institute of Health, 12203 Berlin, Germany; Liliana.HarrisMochmann@klinikum-gap.de (L.H.M.); christiane.spenke@charite.de (C.S.); franziska.briest@charite.de (F.B.); patricia.grabowski@charite.de (P.G.); 2 German Cancer Consortium (DKTK), Partner Site Berlin and German Cancer Research Center (DKFZ), 69120 Heidelberg, Germany; 3 Institute of Pathology, Charité–Universitätsmedizin Berlin, Corporate Member of Freie Universität Berlin Humboldt-Universität zu Berlin and Berlin Institute of Health, 12203 Berlin, Germany; Ruza.Arsenic@patho-diagnostik.ch (R.A.); korinna.joehrens@uniklinikum-dresden.de (K.J.); hedwig.lammert@charite.de (H.L.); michael.hummel@charite.de (M.H.); 4 Institute für histologische und zytologische Diagnostik AG Aarau, 5000 Aarau, Switzerland; 5 Institute of Pathology, Carl Gustav Carus University Hospital Dresden, 01307 Dresden, Germany; 6 Department of Medical Oncology and Pneumology (Internal Medicine VIII), University Hospital Tübingen, 72076 Tübingen, Germany; bence.sipos@med.uni-tuebingen.de; 7 Private Practice of Pathology and Molecular Pathology, 70176 Stuttgart, Germany; 8 iPATH Berlin—Immunopathology for Experimental Models, Core Unit of the Charité, Charité–Universitätsmedizin Berlin, Corporate Member of Freie Universität Berlin, Humboldt-Universität zu Berlin, and Berlin Institute of Health, 12203 Berlin, Germany; Anja.Kuehl@charite.de; 9 Stratifyer Molecular Oncology GmbH, 50935 Cologne, Germany; ralph.wirtz@stratifyer.de; 10 Department of Endocrinology, Universitatsklinikum Erlangen, 91054 Erlangen, Germany; marianne.pavel@uk-erlangen.de; 11 Central Biobank, Berlin Institute of Health, 10178 Berlin, Germany; 12 Department of General and Visceral Surgery, Zentralklinik Bad Berka, 99437 Bad Berka, Germany; Daniel.Kaemmerer@zentralklinik.de; 13 CURANOSTICUM Wiesbaden-Frankfurt in der DKD HELIOS Klinik, 65191 Wiesbaden, Germany; baum@rpbaum.de; 14 Institute of Medical Immunology, Campus Virchow Klinikum, Charité–Universitätsmedizin Berlin, Corporate Member of Freie Universität Berlin, Humboldt-Universität zu Berlin and Berlin Institute of Health, 10117 Berlin, Germany

**Keywords:** ileal neuroendocrine tumors, neuroendocrine carcinoma, immunoprofiling, tumor infiltrating lymphocytes, programmed cell death protein 1, programmed death-ligand 1, tumor-associated macrophages

## Abstract

**Simple Summary:**

Immunotherapy with checkpoint inhibitors targeting the programmed cell death protein 1 (PD-1)/programmed death-ligand 1 (PD-L1) axis or Cytotoxic T-lymphocyte-associated protein 4 (CTLA-4) has shown only modest activity in neuroendocrine tumors (NET) and neuroendocrine carcinomas (NEC). By investigating the tumor immune microenvironment in NET/NEC with immunohistochemistry (IHC) and mRNA immunoprofiling, we found that they lack signs of an activation of an antitumor immune response like intratumoral T cell infiltration and expression of IFNγ regulated genes. But NET and NEC expressed several immunosuppressive genes. This included chemokines, known to attract immunosuppressive myeloid cells but not antitumor effector cells, or genes with immunosuppressive functions. Some of those might be expressed by both tumor cells and immune cells, such as CD74, and represent potential therapeutic targets for immunomodulation.

**Abstract:**

Checkpoint inhibitors have shown promising results in a variety of tumors; however, in neuroendocrine tumors (NET) and neuroendocrine carcinomas (NEC), low response rates were reported. We aimed herein to investigate the tumor immune microenvironment in NET/NEC to determine whether checkpoint pathways like programmed cell death protein 1 (PD-1)/programmed death-ligand 1 (PD-L1) might play a role in immune escape and whether other escape mechanisms might need to be targeted to enable a functional antitumor response. Forty-eight NET and thirty NEC samples were analyzed by immunohistochemistry (IHC) and mRNA immunoprofiling including digital spatial profiling. Through IHC, both NET/NEC showed stromal, but less intratumoral CD3+ T cell infiltration, although this was significantly higher in NEC compared to NET. Expression of PD1, PD-L1, and T cell immunoglobulin and mucin domain-containing protein 3 (TIM3) on immune cells was low or nearly absent. mRNA immunoprofiling revealed low expression of IFNγ inducible genes in NET and NEC without any spatial heterogeneity. However, we observed an increased mRNA expression of chemokines, which attract myeloid cells in NET and NEC, and a high abundance of genes related to immunosuppressive myeloid cells and genes with immunosuppressive functions like CD47 and CD74. In conclusion, NET and NEC lack signs of an activation of the adaptive immune system, but rather show abundance of several immunosuppressive genes that represent potential targets for immunomodulation.

## 1. Introduction

Effector lymphocytes that are found in the tumor microenvironment (TME) of neoplasms with an inflammatory phenotype are often anergic, exhausted, or senescent due to chronic stimulation in this TME. They upregulate inhibitory checkpoint receptors like programmed cell death 1 (PD-1). By binding to its main ligand, PD-L1, which is frequently upregulated on tumor and/or immune cells, proliferation and function of effector lymphocytes is inhibited and a functional antitumor immune response is prevented [1]. T cell immunoglobulin mucin-3 (TIM-3) is another important cancer immune checkpoint that is expressed in different types of immune cells, including T cells, regulatory T cells, dendritic cells, B cells, macrophages, natural killer cells, and mast cells. Like PD-1, TIM-3 has been identified as an important player in CD8+ T cell exhaustion and dysfunction in multiple cancer types in both experimental models and humans [2].

Checkpoint inhibitors are monoclonal antibodies that block these inhibitory molecules. Thereby, they reactivate the function of effector lymphocytes found in the TME. This type of immunomodulation has already reshaped current cancer therapies. PD-1/PD-L1 targeted therapy with checkpoint inhibitors has shown unexpected efficacy in a wide range of metastatic solid tumors with an acceptable toxicity profile [3]. In some patients, checkpoint inhibitors even have the potential to lead to long-term remission. 

Based on these observations and the fact that treatment options in both gastroenteropancreatic (GEP) neuroendocrine tumors (NET) and poorly differentiated neuroendocrine carcinomas (NEC) are limited [4,5], checkpoint inhibitors were also evaluated for NET and NEC in early clinical trials. However, in GEP-NET and NEC only low response rates to anti-PD1 monotherapies were observed [6,7,8]. Combined PD-L1 and CTLA4 blockade demonstrated a 44% overall response rate in patients with nonpancreatic NEC, while in G1/G2 NET objective responses were infrequent [9,10].

Moreover, prediction of patient response to therapy remains a challenge using current methods due in part to the lack of knowledge of NET and NEC tumor immune microenvironment. Therefore, there is a need to develop alternative therapeutic strategies as well as biomarkers for therapy stratification. Several potential predictive biomarkers for response to immune checkpoint inhibition are being evaluated such as determination of PD-L1 expression levels, lymphocyte infiltration, and mutational burden. Efficacy of anti PD-1/PD-L1 treatment seems at least in part to depend on PD-L1 expression [11,12] and T cell infiltration [13,14]. Moreover, antigenicity of the tumor seems to play a role. An important factor that determines the antigenicity is the amount of neoepitopes, which is mainly determined by the mutational load, but also human leukocyte antigen (HLA) heterozygosity [15,16,17,18].

Tumors escape immune surveillance and immune attack by various mechanisms. These escape mechanisms include the loss of tumor antigens and loss or mutation of molecules involved in antigen processing and presentation, rendering themselves invisible to cytotoxic lymphocytes (CTLs) and leading to T cell ignorance [19]. 

Another strategy by which tumors escape the immune system is the induction of tolerance by manipulating the function and proliferation of immune effector cells. This immunomodulation can occur by a variety of mechanisms. Besides expression of inhibitory checkpoint molecules like PD-L1, TIM-3, among others on tumor or stromal cells, this includes active suppression of immune effector cells by secretion of soluble factors like cytokines and growth factors by tumor and stromal cells [20]. These soluble factors promote not only tumor growth but also immunosuppression. They induce T cell and NK cell apoptosis, and block lymphocyte homing, activation, and proliferation. Moreover, they induce and attract cells with immunosuppressive properties, specifically regulatory T cells and immunosuppressive myeloid cells [21,22]. Tumor-associated macrophages (TAMs) are the predominant leukocytes infiltrating the solid tumours and represent the major inflammatory component of the stroma of many tumors. TAM are characterized by the M2 phenotype and favour tumor progression not only by promoting tumor cell proliferation, angiogenesis, matrix remodeling, and tumor migration, but also by suppression of adaptive immunity [22]. 

These different immune escape mechanisms vary between cancer entities and even between tumors of the same entity. Moreover, different escape mechanisms may work simultaneously. 

To date, the immunophenotype and immune escape mechanisms of NET and NEC are not well defined. Different studies showed infiltration of CD3+ T cells in GEP-NET [23,24,25]. In neuroendocrine neoplasia (NEN) G3, comprising well differentiated NET G3 and poorly differentiated NEC G3 (each with a higher proliferation rate of ki67 > 20%) infiltration of CD3 T cells could be observed in 45.5% and 97% of cases [23,26]. In intermediate grade NET, a correlation between high numbers of CD3^+^ T cells and a longer recurrence-free survival was observed. Meanwhile, a high infiltration of CD3^+^FOXP3^+^ regulatory T cells in poorly differentiated, metastatic NEC seemed to be predictable for a shorter overall survival of patients [23]. Moreover, the extent of macrophage infiltration was reported to be higher in NEC than in NET [27] and is predictive for recurrence following surgery [28]. In GEP-NEN of different localizations, PD-L1 expression was significantly associated with a high-grade WHO classification [26,29,30]. 

Gaining a deeper understanding of TME through immunoprofiling may aid in the stratification of patients for different immunotherapeutic approaches. Immunoprofiling of various solid tumors such as melanoma, lung cancer, and renal cell cancer has been a useful tool to predict treatment response to immunotherapy like checkpoint blockade, and also to identify new immunotherapeutic targets [31]. Here we analyzed the TME of well-differentiated NET and NEN G3 by immunohistochemistry (IHC) staining for CD3, PD-1, PD-L1, and TIM-3, and present to our knowledge for the first time detailed mRNA immunoprofiling including spatially resolved, high-plex mRNA digital profiling. 

We found that well-differentiated NET and NEN G3 lack signs of an activation of the adaptive immune system, but rather display a gene signature associated with infiltration of myeloid cells including high expression of several immunosuppressive genes known to be expressed by tumor cells or stromal cells.

## 2. Results

### 2.1. Patient Characteristics

The most important clinicopathological characteristics of the patients from samples used for immunohistochemistry and mRNA profiling are shown in Table 1. Detailed characteristics of each patient including the Tumor-Node-Metastasis (TNM) stage and metastasis localization are listed in Table S1. A total of 78 patients diagnosed with NEN were enrolled in our study: 48 gastroenteropancreatic (GEP)-NET G1/G2 and 30 NET G3/NEC of different localizations. Fifty-six patients had distant metastasis, 2 patients had a history of or were diagnosed with a local disease of a secondary cancer during follow-up. Treatment strategies were known for 61/75 patients. Among these, 15 NET G1/G2 patients underwent surgical resection. Medical treatment was performed in 42 patients, including somatostatin analogs, interferon α, mTOR inhibitors, chemotherapy, and targeted radionuclide therapy (PRRT). Three patients with NET G1/G1 received somatostatin analogs prior to tissue collection. One of the patients received interferon α for one year; however, tumor tissue for analysis was collected eight years thereafter. All patients with NET G3/NEC underwent diagnostic biopsy and received chemotherapy, and two of them received chemotherapy prior to tissue collection. Chemotherapy was stopped at least 12 weeks before tissue collection. 

### 2.2. Lower Intratumoral CD3+ T Cell Infiltration in NET G1/G2 Compared to NETG3/NEC 

**Figure 1:**
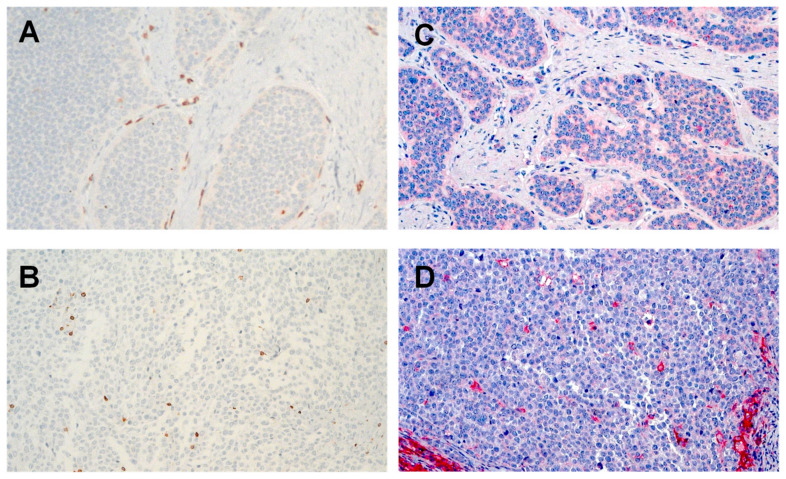
Immunohistochemical staining. (**A**) CD3 in neuroendocrine tumors (NET) G1/G2 with stromal T cell infiltration 8/1 high-power fields (HPF); (**B**) CD3 in NET G3/neuroendocrine carcinomas (NEC) with intratumoral T cell infiltration 10/1 HPF; (**C**) programmed death-ligand 1 (PD-L1) in NET G1/G2, weak staining in >1% tumor cells; (**D**) PD-L1 in NET G3/NEC; strong staining >1% of tumor cells original magnification × 300.

Based on the hypothesis that the tumor immune microenvironment of well-differentiated NET G1/G2 differs from NEN G3, we compared CD3 infiltration in NET G1/G2 with that in NET G3/NEC (NEN G3). For an initial exploratory analysis of CD3 expression, tissue microarray slides containing samples of 25 NET G3/NEC as well as 47 NET G1/G2 of two different institutions (Charité Berlin and Zentralklinik Bad Berka) were stained for CD3 expression. CD3+ lymphocytes were seen in greater numbers in NET G3/NEC (median number 70/high-power fields (HPF), range 8–300) than in NET G1/G2 (20/10 HPF, range 2–140). To analyze CD3+ T cell infiltration in more detail with regard to stromal and intratumoral T cell infiltration and to account for potential tumor heterogeneity, whole slides of 29 NET G3/NEC and 41 NET G1/G2 were available and evaluated for stromal and intratumoral CD3+ T cell infiltration (Figure 1A,B, Table 2). There was no significant difference in stromal CD3+ T cell infiltration between NET G1/G2 (median cell number 60/10 HPF, range 25–87) and NET G3/NEC (median cell number 58/10 HPF, range 18–150, *p* = 0.54). However, NET G3/NEC showed a significant higher intratumoral CD3+ T cell infiltration than NET G1/G2 (median cell number 16/10 HPF, range 3–75 vs. 9/10 HPF, range 4–51; *p* = 0.0001, Mann–Whitney U test). 

### 2.3. PD-L1 Is Expressed Higher in NET G3/NEC than NET G1/G2

Whole slides of NET G3/NEC and NET G1/G2 were analyzed for PD-L1 expression on tumor cells and stromal cells by IHC. Staining was defined as positive if the cell membrane displayed PD-L1 expression levels of >1% (Figure 1C,D, Table 2). PD-L1 expression could be more frequently detected in NET G3/NEC than in NET G1/G2: 11/46 NET G1/G2 (24%) and 15/30 NET G3/NEC (50%) were classified as PD-L1 positive (*p* = 0.0001; Mann–Whitney U Test). PD-L1 was predominantly expressed by tumor cells. 

### 2.4. Low PD-1 and TIM-3 Expression on Stromal and Intratumoral T Cells 

In only two out of 17 samples of NET G3/NEC, PD1+ lymphocytes could be detected, whereas no PD-1 positive lymphocytes could be detected in 42 samples of NET G1/G2 (Table 2). 

Therefore, we evaluated intratumoral and stromal TIM-3 expression by IHC. TIM-3 expression could be detected on stromal lymphocytes in 16/23 NET G3/NEC and in 20/36 NET G1/G2. However, median counts/10 HPF were low in NETG1/G2 (4.2 (range 0–20)) and in NET G3/NEC [3.7 (range 0–16)]. Low intratumoral expression could be detected only in 1/36 NET G1/G2 (5/10HPF) and 5/23 NET G3/NEC (3–5/10 HPF) (Table 2).

### 2.5. mRNA Immunoprofiling Showed Differential Immune Response in NET G1/G2 and NET G3/NEC 

NanoString^®^ gene expression analysis using the PanCancer^®^ Immune Profiling Panel (NanoString Technologies, Seattle, WA, USA) was performed with mRNA of 26 NET G1/G2 samples, 25 NET G3/NEC samples, and 14 healthy control samples (ileum mucosa and submucosa tissue) of patients not suffering from NEN. 

Immunohistochemistry data and qRT-PCR data of PD-1 and TIM-3 were confirmed: Counts for both PD-1 and HAVCR coding for TIM-3 were low, as well as counts for molecules known to be upregulated in anergic and/or exhausted T cells (LAG3, TNFRSF9, HAVCR2, ALCAM, TIGIT, CTLA4, TNFRSF1B, CCL4, CD200, OX-2, NRP1, IL21, and CD28 or transcription factors including EGR2, NFATC1, and IRF4). 

Analysis of differentially expressed genes (Table 3) revealed that 9 genes were significantly (false discovery rate (FDR) < 0.05) upregulated and 6 were significantly downregulated compared to healthy tissue in NET G1/G2. This included mainly genes involved in innate immune response. This applies also to NET G3/NEC when comparing to healthy ileal tissue. In NET G3/NEC, 4 genes were significantly upregulated and 6 genes were downregulated. Some genes were only expressed significantly different in NET G1/G2 or NET G3/NEC than in healthy tissue. Unfortunately, comparison to a healthy organ site matched control was not possible due to paucity of material. 

**Figure 2:**
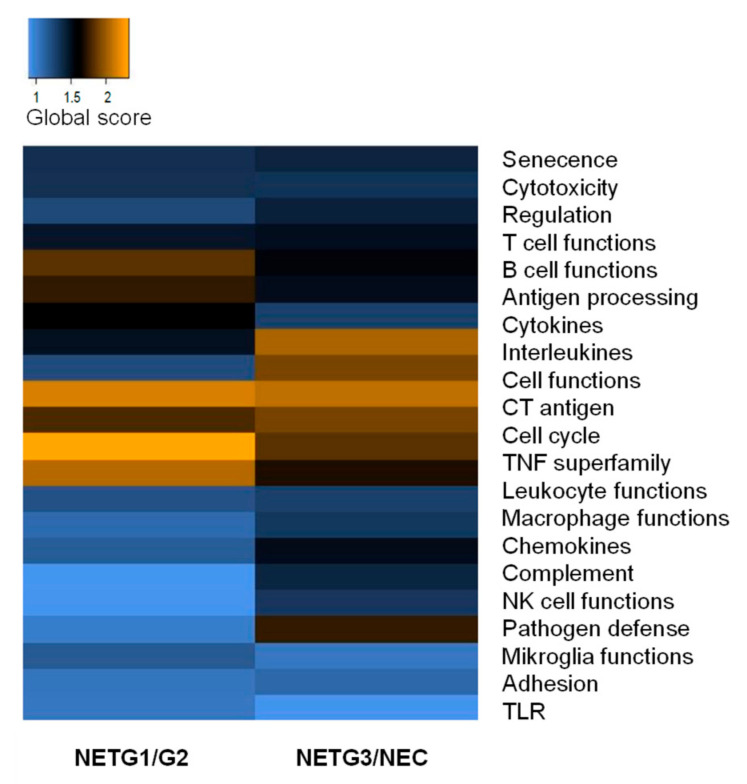
Differential expression at the gene set level in NET G1/G2 and NET G3/NEC. For each gene set the most differentially expressed genes were compared to healthy tissue and the extent of differential expression in each gene set was summarized using a global significance score.

Next, the most differentially expressed genes were clustered to gene function. The extent of differential expression in each gene set was summarized using a global significance score (Figure 2). Cancer testis genes and genes associated with cell function and cell regulation, cell cycle, and senescence were differentially expressed compared to healthy tissue, reflecting the abundance of tumor cells. Gene sets associated with immune cell function revealed that genes associated with T cell function and NK cell function and genes associated with cytolytic activity like GZMA, GZMK, and PRF1 and antigen presentation (PSMB7, HLA-DMB) were lower expressed in NET G3/NEC and NET G1/G2 compared to healthy tissue. TNF receptor family genes and genes associated with chemokines, interleukins, and cytokines were differentially expressed in NET and NEC compared to healthy ileal tissue: Most of the genes associated with these gene sets showed a lower expression level reflecting the lower abundance of immune cells in tumor samples. Compared to healthy ileal tissue, mRNA expression of CD45 was lower in NET G3/NEC and NET G1/G2 (*p* < 0.001). However, genes known to be involved in cancer sustenance and progression such as TNFRSF11B, IL8, CXCL2, CXCL16, SPP1, IL1RAP, MIF, and VEGFA showed higher expression levels in NET G3/NEC and NET G1/G2 compared to healthy tissue. 

**Figure 3:**
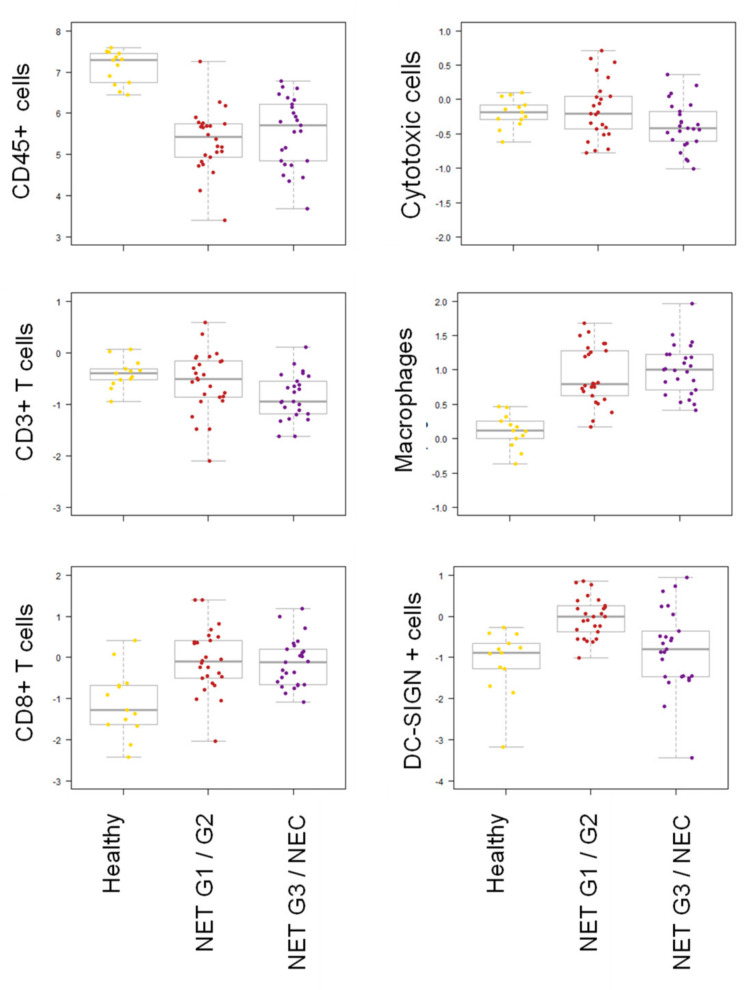
Differential expression of genes associated with cell types in NET G1/G2 and NET G3/NEC. Cell scores (cell type/tumor infiltrating immune cells) of different immune cell subsets were calculated by the expression of mRNA markers specific for each cell type relative to CD45 mRNA levels: T cells, CD3D, CD3E; CD8+ T cells, CD8A; cytotoxic cells, PRF1, KLRK1, KLRB1, GNLY, GMZA; macrophages, CD84, CD163, CD68; DC-SIGN positive cells, CD209/DC-SIGN.

Indeed, when calculating cell scores of different immune cell subsets by the expression of mRNA markers specific for each cell type relative to CD45 mRNA levels (cell type/tumor infiltrating immune cells), scores for T cells (CD3D, CD3E) and cytotoxic cells (PRF1, KLRK1, KLRB1, GNLY, GMZA) were marginally lower, but cell scores for CD8+ T cells (CD8A) and macrophages (CD84, CD163, CD68) were higher (Figure 3). Interestingly, in NET G1/G2 and NET G3/NEC we also observed higher expression of CD209/DC-SIGN, a marker for dendritic cells but also for tumor-associated macrophages (TAMs) and immature immunosuppressive myeloid cells [23]. This suggests an infiltration of both NET and NEC by cells of both the innate immune system and the adaptive immune system including CD8+ T cells with a quantitative and qualitative difference compared to healthy ileal tissue.

### 2.6. Digital Spatial Profiling of NEC and NET G3 Samples

In recent years, it has become clear that the NEN G3 group represents a heterogenous group of NET G3 and NEC with different molecular and clinicopathological properties. Therefore, we asked whether the tumor immune microenvironment differs in these two groups. We performed GeoMx® NanoString Digital Spatial RNA Profiling (NanoString Technologies, Seattle, WA, USA) on two microarray slides consisting of 12 NEC and 18 NET G3 to determine immune signatures within tumor regions and to get more insight into the immune contexture and spatial heterogeneity. Patients’ characteristics of this cohort are depicted in Table 4.

**Figure 4:**
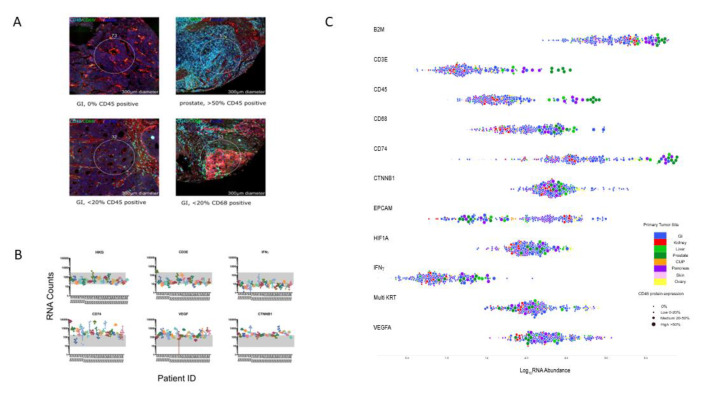
Digital spatial profiling (DSP) tumor regions were scanned for detection of immune cells in NET G3/NEC tumors. (*A*) Fluorescent labeling aided in characterizing tumor versus immune cells that included hematopoietic marker CD45 (cyan) and monocyte/macrophage marker CD68 (green), NET/NEC tumor marker, synaptophysin (red), and DNA (blue). Representative images portray the varying levels of leukocytes (CD45 and CD68) within the tumors (gastrointestinal (GI) and prostate) and show staining for the immune cells within the regions of interest (ROIs) in a selected 300-µm width (circled). (*B*) The distribution of RNA abundance in key genes for each patient surveyed in this study. Four to six ROI were selected for each patient. The geometric mean of 5 housekeeping proteins (HKG) was used as normalization and as a baseline reference for low and high RNA abundance. Highlighted are the low DSP RNA abundance for IFNγ and CD3E genes and high DSP RNA abundance for CD74, VEGFA, and CTNNB1. (*C*) Swarm blot to stratify the RNA abundance of high and low expressed genes relative to the scoring of ROIs with high CD45 positive cells (indicated by circle size). To determine the relative amount of CD45 positive cells within an ROI, the immunofluorescent 300-µm scanned regions were qualitatively scored. In the legend, the colors represent the tumor site.

Regions of interest (ROI) were selected based on Ki67 positivity (tumor-enriched regions) and immunofluorescence imaging of CD45, CD68, and tumor marker synaptophysin expression (Figure 4A) to scan tumor regions. RNA counts for each 85-gene probe set were then quantified by the nCounter platform. Within the 85-gene RNA probe set are specific gene signatures that define genes associated with certain immune responses, thus allowing the identification of immune phenotypes across NET/NEC samples. 

**Figure 5:**
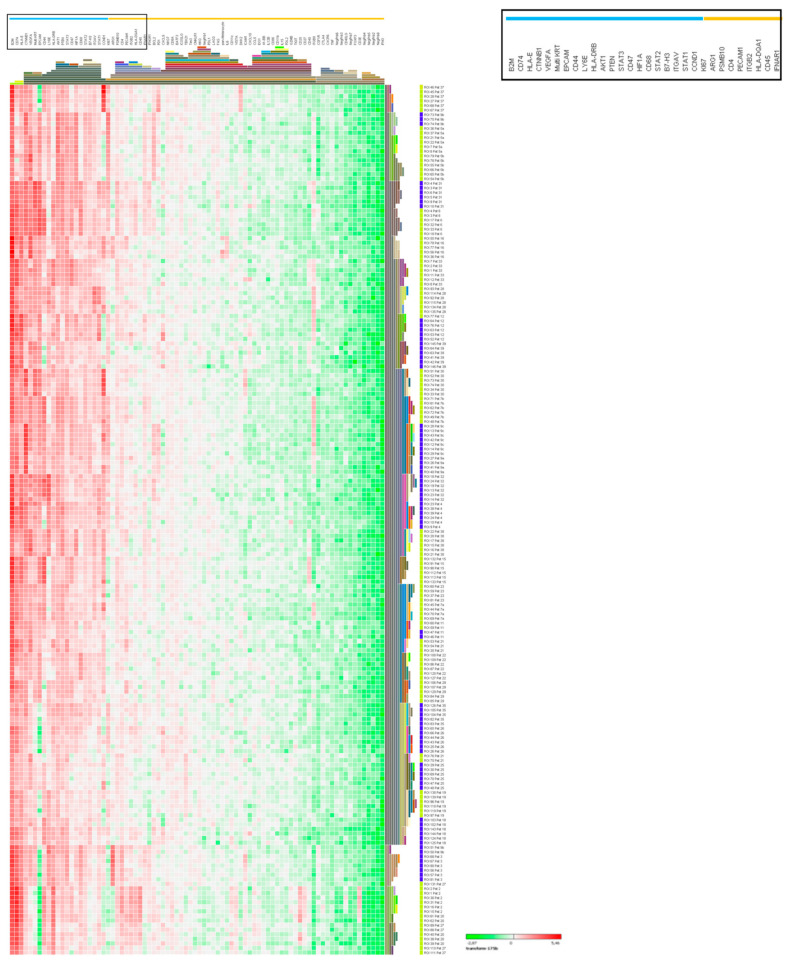
RNA abundance heatmap of genes related to the immune response in NET G3 and NEC. The columns represent the log10 transformation of 85 gene probes. The red to green hues represent the gradient high to low RNA abundance. The values used are normalized to the geomean of 5 housekeeping proteins. Rows represent ROI ID and the bar on the right is color coded for NEC (yellow) and NET G3 (blue). The hierarchical gene clustering shows the high to low RNA abundance.

Immunofluorescence of 30 NET G3/NEC patients showed overall varied expression of synaptophysin, CD45, and CD68 as expected with such heterogenous tissues derived from distinct tumor sites, morphologies, and disease stages. Despite these distinct tumor types, evaluation of RNA counts across samples for CD45 correlated with CD3E and with CD68 (*p* < 0.0001) (Figure 4B,C). An independent pathologist scored the fluorescence signal for CD45 expression to determine a correlation between immunofluorescence (% positive staining) and CD45 RNA counts, and indeed these changes correlated in a qualitative manner (Figure 4C). The same was true for CD68 immunofluorescence and CD68 RNA and CD45 immunofluorescence staining correlated qualitatively with CD68 immunofluorescence but not synaptophysin immunofluorescence. Moreover, genes from housekeeping controls (average of 6-probe set), low (CD3E and IFNg), and high (CD74, VEGF, and CTNNB1) expressed genes were plotted to determine if RNA count signals were specific to the patient and not false positive errors introduced by fixation or handling. Indeed, high count genes such as CD74, VEGF, and CTNNB1 were significantly expressed above housekeeping gene levels and were patient-specific (Figure 4B). Following normalization, gene expression patterns from the 85-gene RNA probe were obtained from unsupervised hierarchical clustering. Overall, we found that RNA expression of each ROI clustered by patient, including three patients from whom primary tumor and matched metastases were analyzed. Genes also clustered by function. Moreover, further clustering analysis resulted in a two cluster pattern of high and low expression (Figure 5). Interestingly, NETG3 and NEC did not cluster, indicating that high and low expressed genes are independent of disease pathology. In addition, gene expression was independent of disease localization. This result may be due to the several tumor sites screened; thus, a greater cohort of a single tissue site may correlate with the disease grade. Interestingly, both immune cell and tumor cell in the ROIs, low abundance of CD3E and IFNγ, plus IFNγ signature genes were observed (Figure 4B,C and Figure 5). In contrast, genes with immunosuppressive functions like VEGFA, HIF1α, CD47, CD74, and CD44, as well as signaling pathway-associated molecules like AKT, PTEN, STATs, and CTNNB1 coding for PI3K and WNT signaling, were highly expressed in both tumor cell (EPCAM and MultiKRT) and immune cell (CD45)-enriched regions (Figure 4B and Figure 5). Moreover, high CD74 co-expression with immunosuppressive genes VEGFA and HIF1α and hematopoietic marker CD45 and tumor marker MultiKRT demonstrates that CD74 is expressed by both the tumor cells and the immune cells. CD74 is an HLA class II protein regulating dendritic and macrophage inflammation and is a receptor for macrophage migration inhibitory factor (MIF). CD74 activation induces PI3K-AKT signal transduction and has a role in inflammatory diseases, and thus may be relevant in targeting an immunosuppressive TME of NET/NEC. 

## 3. Discussion

We hypothesized that the tumor immune microenvironment of NET G1/G2 differs from poorly differentiated NEN. Therefore, we analyzed the TME of NET G1/2 and NET G3/NEC by IHC and mRNA immunoprofiling including digital spatial profiling using NanoString^®^ technology. We found that NET G1/G2 and NET G3 are characterized by a different immune contexture and signature. But both NET G1/G2 and NET G3/NEC showed low expression of IFNγ-associated genes and low intratumoral T cell infiltration. 

Besides T cell infiltration, PD-L1 expression on the surface of both tumor cells and immune cells has been identified as an important factor in response to immunotherapies targeting the PD-1 axis [11,12]. The ability of tumors to induce an adaptive immune response that in turn leads to the expression of inhibitory checkpoint molecules such as PD-L1 on immune cells and tumor cells is a necessary requirement for their effectiveness. These tumors are so called “T cell inflamed” or “hot” tumors and are characterized by an intratumoral infiltration of exhausted T cells, infiltration of immunosuppressive cells such as regulatory T cells and myeloid cells, and expression of IFNγ inducible genes related to an adaptive immune response [14]. 

In our set of ileal NET G1/G2 and NET G3/NEC of different origins, PD-L1 protein expression was detected in 24% of NET and 50% of NET G3/NEC as determined by IHC and was preferentially expressed on tumor cells. An increase in PD-L1 expression with grading of NEN and preferential expression on tumor cells was also observed in other studies of GEP-NEN [29,32]. 

However, Ferrata et al. observed staining on tumor cells and immune cells in their GEP-NEN cohort [26]. 

Immunohistochemistry (IHC) staining revealed, although low, intratumoral CD3+ T cell infiltration and mRNA immunoprofiling of whole tumor slides, suggesting infiltration with CD8+ T cells as shown by others by IHC in GEP-NEN [26,33]. However, these T cells seemed not to be activated. In accordance with results of previous studies in GEP-NEN [26,32,33,34,35], we observed low expression of the exhaustion marker PD1 on T cells. We expanded the analysis to include IHC staining for TIM-3, another exhaustion marker, which was also low in both NET G1/G2 and NET G3/NEC. Furthermore, NanoString^®^ immunoprofiling revealed low expression levels of genes related to an induction of an adaptive immune response like IFNγ inducible genes [36], especially genes coding for T cell exhaustion proteins, confirming IHC results. Considering the low intratumoral T cell infiltration and lack of an IFNγ immune signature related to an adaptive immune response, including missing expression of T cell exhaustion markers, PD-L1 expression in NET G1/G2 and NET G3/NEC might be tumor-intrinsic and regulated by oncogenic pathways like MAPK, PI3K/PTEN, and p53, rather than reflecting an immune active tumor microenvironment [37,38,39]. Therefore, PD-L1 expression is unlikely to be predictive for response to the PD-1 blockade in NET and in NEC. 

Although the assessment of PD-L1 and PD-1 expression on tumor and immune cells can be useful to predict clinical response to PD-1 checkpoint blockade, recent studies have shown that it might be only one part that is relevant for a favorable outcome under the PD-1/PD-L1 blockade [31,40] 

Tumor and host immune cell interactions within the tumor immune microenvironment are more complex. One important factor is the spatial localization of T cells. In two recent studies of GEP-NETs, intratumoral T cell infiltration was reported to increase with grade [32,41]. Moreover, Cives et al. observed a significant higher extent of lymphocyte infiltration in duodenal NET as compared with jejunal or ileal NET [33]. We found in our set of NEN intratumoral infiltration of T cells slightly higher in NET G3/NEC as compared to NET G1/G2 that were mainly of ileal origin. 

However, in both NET and NET G3/NEC, intratumoral T cell infiltration was low; T cells could be detected preferentially in the tumor stroma. Exclusion of T cells from the proximity of cancer cells was also found in other solid cancers [42]. 

Abundance of immunogenic cancer rejection antigens, especially neoantigens, have been proposed as a critical determinant for induction of a tumor-specific T cell response [15,16,43]. Higher mutational load correlated positively with immunogenicity and thus T cell infiltration and was a predictor for clinical response to anti-PD-1 inhibition in non-small cell lung cancer (NSCLC) [15]. NET have only a low mutation rate. Therefore, one might speculate that the low intratumoral infiltration and lack of T cell activation in NET might be related to a lower neoantigen burden compared to NSCLC. Poorly differentiated NEC have a higher mutation rate compared to NET and thus should be more immunogenic [44]. Although intratumoral T cell infiltration was slightly higher in NEC compared to NET, an active immune response represented by an IFNγ signature was lacking in both NET and NEN G3. However, mutational load did not correlate in all tumor types with IFNγ signature [16] or T cell infiltration [45]. NEC carry p53 mutations and it has been shown that p53 mutant tumors display only low levels of genes associated with cytotoxic immune activation [46].

Several mechanisms might lead to the failure of T cell activation. Besides the lack of proximity to cancer cells, this includes defects in priming of T cells in the tumor-draining lymph nodes, which has not been analyzed in this study, or loss of antigen presentation. Defects of the antigen processing machinery (APM) or loss of HLA expression, as reported for pancreatic NET [28], is unlikely as we observed no significant downregulation of APM molecules or HLA class I molecules on mRNA levels compared to healthy ileal tissue. However, defects due to epigenetic regulation cannot be completely excluded. 

We found genes coding for chemokines and cytokines attracting myeloid cells as well as coding for macrophages/myeloid cell markers higher expressed in NEN compared to healthy ileal tissue. For NET G3 and NEC we confirmed macrophage infiltration by CD68 immunofluorescence staining. Our results are supported by previous studies showing infiltration of macrophages in pancreatic NEN [27].

These cells can induce an immunosuppressive microenvironment and are associated with cytokines such as TNFα, VEGFA, and IL8, and accumulate in hypoxic regions of the tumor [47,48]. 

We could identify high expression of several genes with immunosuppressive functions like *VEGFA*, *HIF1a, HLA-E, CD47*, *CD74,* and *STAT3* in NEC and NET G3. Some of these genes are target genes of the WNT pathway like *CTNBB1*, *CD44*, *VGFA*, and *CD47,* or are regulated by hypoxia such as *STAT3*, *HIF1A*, *VEGFA,* or *HLA-E*. 

Interestingly, we observed high mRNA expression of the signaling molecules *AKT*, *PTEN*, *STATs,* and *CTNNB1* coding for beta-catenin in tumor and immune cell regions of both NET G3 and NEC. Associated pathways can be active in immunosuppressive cells such as tumor associated macrophages (TAM) or myeloid-derived suppressor cells, but also in tumor cells of NEN [44].

mRNA immune profiling and immunofluorescence data therefore rather indicate tumor intrinsic mechanisms, leading to infiltration of myeloid cells that contribute not only to angiogenesis and tumor progression but to establishment of a chronic inflammatory, immunosuppressive immune contexture and in turn to T cell exclusion [42].

Enhanced or aberrant activation of pathways like the PI3K/PTEN/AKT pathway [49,50,51] and WNT/β-catenin pathway [52,53] or constitutive STAT3 signaling [54] has been shown to play a role in NEN, especially NET, but has also been identified as an immune escape mechanism, leading to reduced cell infiltration within the tumor immune microenvironment [55,56]. Activation of the PI3K/PTEN/AKT pathway has been shown to lead to recruitment of TAMs [57]. In a mouse model it has been shown that STAT3 expression leads to reduced recruitment and activation of T cells by decreased expression of proinflammatory mediators, including the chemokines CCL5 and CXCL10 [58]. Activation of the WNT/β-catenin pathway downregulates CCL4 expression by tumor cells and thereby prevents cross-priming of antitumor T cells due to failed recruitment of CD103 + dendritic cells [59]. 

Hence, these pathways are potential targets to enable intratumoral T cell infiltration and therefore candidates for the development of immunotherapeutic combination therapies, i.e., with checkpoint inhibitors, at least for poorly differentiated NEC G3. 

Although we observed low intratumoral T cell infiltration and a lack of INFγ signature in both NET and NEC, but rather the expression of genes that are associated with myeloid cell infiltration, we still found differences in gene expression between NET G1/G2 and NET G3/NEC. This applied not only to gene sets related to cell function, but also to gene sets related to interleukins, cytokines, and chemokines, which showed varied differential expression compared to healthy ileal tissue. This might reflect the molecular differences between well and poorly differentiated NEN and their impact on the tumor immune microenvironment. p53 and Rb1 mutations are pivotal drivers of NEC of any anatomical origin but are lacking in NET. Hallmarks of NET G1/G2 are loss of chromosome 18, inactivation of CDKN1/APC, and gain of chromosomes 4, 5, and 14 in NET of the small intestine and inactivation of MEN1/chromosome 11, DAXX/ATR, and PTEN/TSC2 in pancreatic NET [34].

Interestingly, gene expression analyses by DSP profiling revealed that NEC and NET G3 did not cluster separately, but showed a similar gene expression profile, although NEC and NET G3 have to be regarded as different neoplasms based on their different clinicopathological properties and genetic background. NET G3 bear anatomic site-specific alterations that are a hallmark of NET G1/G2 [34]. Moreover, DSP revealed that different regions of each patient sample showed similar gene expression patterns, and spatial tumor heterogeneity seemed not to matter here. In addition, in the three patients of whom primary tumor and metastases could be analyzed, we observed a similar gene expression pattern as well. 

## 4. Materials and Methods 

### 4.1. Patients

Seventy-eight patients diagnosed with GEP-NET and NEC in different localizations were enrolled in the study. The tissue samples were collected from three German hospitals: Charité–University Hospital, Berlin, Zentralklinik Bad Berka GmbH, and University Hospital Tübingen. All samples were classified according to the WHO Classification of GEP-NEN (2010). Poorly differentiated NEN were reclassified according to WHO classification 2019 in NET G3 and NEC [60], and the pancreatic NET/NEC were re-classified and adapted according to the new WHO classification (2017) [61].

Samples were obtained before any therapy (NEC) or at least 12 weeks after the end of any tumor-specific therapy including biotherapy, chemotherapy, interferon α, or targeted therapy (NET). 

The treatment response was evaluated at baseline and every 12 weeks thereafter or, as clinically indicated, by CT scans or MRI of the chest, abdomen, and brain following Response Evaluation Criteria In Solid Tumors (RECIST) criteria [62].

Fourteen samples of healthy ileum mucosa and submucosa tissue from patients not suffering from neuroendocrine neoplasms (NEN) could be collected for comparison with NEN. Approval by the ethical institutional review board of the Charité–Universitätsmedizin Berlin for investigation of prognostic and immunologic factors was obtained (reference number EA1/325/14, date: 21 November 2014).

### 4.2. Immunohistochemistry

Tissue microarrays (TMA) and whole slides were stained for CD3+ (T cells, Agilent; clone A0452, dilution 1:200), PD-1 (eBioscience, Thermo Fisher Scientific Inc., Waltham, MA, USA, clone J121; dilution 1:100), TIM-3 (LSBio, Seattle, WA, USA, aa 176–194, dilution 1:200), and PD-L1 (Abcam, Cambridge, UK, #ab58810 dilution 1:300; E1L3N Cell Signaling, Danvers, MA, USA, dilution 1:100). Human tonsil formalin-fixed paraffine-embedded (FFPE) tissues with and without primary antibody were used as positive and negative controls, respectively, with each run of IHC staining. TMAs contained three cores of each NEN that were selected randomly from areas showing high cellular density. Necrotic areas were avoided. Each core had a diameter of 1 mm. The total surface area of three cores is 2.35 mm^2^. Tissue on whole slides covered an area of 9–100 mm^2^. On whole slides, CD3 T cell infiltration was separately analyzed in the stromal and intratumoral compartment. PD-L1 staining on tumor or immune cells was defined as positive if the cell membrane displayed PD-L1 expression levels of >1%. Two independent reviewers (R.A. and P.G.) evaluated the results and counted the number of positive cells for each antibody. 

### 4.3. Extraction of RNA

Tumor areas were identified by a pathologist on all hematoxylin and eosin (HE)-stained tissues. Afterwards, macrodissection was performed on FFPE-material, leaving only tumor tissue for extraction of RNA. RNA isolation from FFPE tissues was conducted using the ExpressArt FFPE Clear RNAready Kit (AmpTec, Hamburg, Germany) in accordance with the manufacturers’ instructions. Qubit fluorometric quantitation (Thermo Fisher Scientific Inc., Waltham, MA, USA) was used for quantification of RNA concentration. 

### 4.4. mRNA Immunoprofiling (NanoString)

Gene expression analysis was performed using the PanCancer^®^ Immune Profiling Panel of NanoString^®^ nCounter Technology (NanoString Technologies, Seattle, WA, USA) in accordance with the manufacturer’s instructions. NanoString^®^ technology counts fluorescent barcode probes to determine the absolute number of mRNA-transcripts. The panel that was used for the study contains probes for 770 different transcripts analyzing immune cells and categories of immune response [63]. 

### 4.5. Digital Spatial Profiling 

GeoMx®NanoString Digital Spatial RNA Profiling (DSP) (NanoString Technologies, Seattle, WA, USA) was used to unveil the immune landscape of NEC/NET G3. Two tissue microarray slides (STMA15 and STMA16) were incubated with fluorochrome-labeled antibodies against synaptophysin (red), CD45 (cyan), and CD68 (green). Nuclei were stained with DAPI (blue). The selection of regions of interest (ROI) was based on Ki-67-positive immunohistochemistry of both STMA15 and 16; the Ki-67 cut-off value was >20% for NET G3 and >55% for NEC. These ROI coordinates were overlaid and a near match to the immunofluorescent-stained slides. Four to six ROIs were selected for each patient sample. Digital counts from 85 UV-photocleavable RNA probes were counted in the tissue regions exposed to UV light. The RNA counts were from the ultraviolet light-masked area, which was 300 µm2 in size. The RNA abundance for each gene was normalized to the geomean of 5 housekeeping targets (RAB7A, UBB, OAZ1, POLR2A, SDHA), and 8 negative control probes (non-transcript binding) were used to account for background and technical variability. 

### 4.6. Statistical Analysis

The statistical analysis of immunohistochemistry data and qRT-PCR data was performed using IBM® SPSS® Statistics software (release 22.0, IBM Corporation, Armonk, NY, USA). All tests were two sided, and statistical significances were assumed if the null hypothesis could be rejected at the *p* < 0.05 level. The analysis has to be regarded as exploratory, since no correction for multiple testing was applied. 

Analysis of NanoString mRNA expression data was performed with the nSolver™ software version 4.0 using the Advanced Analysis Module version 2.0 (NanoString Technologies, Seattle, WA, USA). Data were normalized using the popular geNorm algorithm to identify an optimal subset of the built-in control gene probes for normalization [64]. Then, a log2 transformation was performed to approximate normal distribution.

Estimated log2 fold-differences in gene expression representing the average magnitude of a gene’s differential expression were calculated by first principal component (PC) analysis followed by multivariate linear regression including adjustment for age, sex, and binding density. To correct for multiple comparisons, the Benjamini–Yekutieli false discovery rate (FDR) was calculated. Statistical significance was assumed at the FDR < 0.05 level.

Pathway dysregulation was scored within the nSolver^™^ software using Gene Set Analysis (GSA) with healthy ileal tissue as the baseline reference. Global significance statistics were also calculated for each pathway by measuring the cumulative evidence for the differential expression of genes in a pathway. 

Comparisons of cell scores calculated by the nSolver^™^ software were done using SPSS® Statistics software.

## 5. Conclusions

Collectively, our analyses led to the conclusion that NET and NEC lack signs of an activation of the adaptive immune system like PD-L1 expression on immune cells or intratumoral infiltration of T cells expressing T cell exhaustion markers like PD-1 or TIM-3. Rather, they display a gene signature associated with infiltration of myeloid cells including high expression of several immunosuppressive genes known to be expressed by tumor cells or stromal cells. Therefore, successful immunotherapy for GEP-NET and NEC requires modulation of the tumor immune microenvironment to enable T cell infiltration into the tumor and induction of a T cell immune response that then might be combined with checkpoint inhibitors to restore activation of exhausted T cells. Further studies are necessary to elucidate in detail the molecular mechanisms and pathways that impair intratumoral infiltration and activation of T cells for the development of combination immunotherapeutic strategies. 

We identified several immunosuppressive molecules, which are already targetable by approved drugs such as bevacizumab directed against VEGF or for which drugs are in development. Besides STAT3 inhibitors, this includes inhibitors of CD47, a “don’t-eat-me” signal that inhibits macrophage phagocytosis for immune evasion [65] or the blockade of MIF–CD74 signaling on myeloid cells to decrease the expression of immunosuppressive factors from myeloid cells and to increase the capacity of dendritic cells to activate CTL [66]. Before evaluation of combination therapies, however, the expression at the protein level should be confirmed as we have already shown for STAT3 [54].

## Figures and Tables

**Table 1:** Patient characteristics for samples used for immunohistochemistry and mRNA profiling.

	*N*
**Patient samples available**	78
**Sex available**	73
Male/female	37/36
Median age	64 (30–84)
**WHO grading classification available**	78
NET (G1/G2)	48 (36/12)
NEC/NET G3	30 (20/10)
**Stage at time of tissue collection**	
NET (G1/G2)	
II/III/IV	1/9/38
NEC/NET G3	
II/III/IV	6/7/17
**Primary tumor localization (*n*)**	
NET (G1/G2)	Ileum (47), pancreas (1)
NEC/NET G3	Gastrointestinal (17), bile duct (3), gall bladder (1), pancreas (5), bladder (1), kidney (1), CUP (2)
**Tissue analyzed Primary tumor/metastasis**	
NET (G1/G2)	48/0
NEC/NET G3	24/6
Localization of analyzed metastasis	Lymph node (3), liver (2), skin (1)

CUP, carcinoma of unknown primary.

**Table 2:** Immunohistochemistry results in NETG1/2 and NET G3/NEC.

	NET G1/G2	NET G3/NEC
**CD3+ T cell infiltration**		
Patient samples available	41	29
Stromal T cell infiltration Median counts/10 HPF	60 (range 25–87)	58 (range 18–150)
Intratumoral T cell infiltration Median counts/10 HPF	9/10 HPF (range 4–51)	16/10 HPF (range 3–75)
**TIM-3 expression**		
Stromal lymphocytes Positive samples	20/36 (56%)	16/23 (70%)
Intratumoral lymphocytes Stained positive	1/36 (3%)	5/23 (22%)
**PD-1/PD-L1 expression**		
PD-L1-Expression on tumor cells Expression level >1% on cell membrane	11/46 (24%)	15/30 (50%)
PD-1-Expression on lymphocytes Expression level >1% on cell membrane	0/42 (0%)	2/17 (12%)

**Table 3:** Differentially expressed genes compared to healthy ileal tissue.

Genes	Log2 Fold Change	Std Error (log2)	*p*-Value	BY *p*-Value	Annotation
**NET G1/G2**					
**MME**	−10.9	1.83	1.73 × 10^−7^	0.000178	Cell functions, cell type-specific
**ABCB1**	−5.3	0.968	9.42 × 10^−7^	0.000487	CD molecules (MDR)
**CASP1**	−4.23	0.725	2.41 × 10^−7^	0.000187	Innate immune response
**MAF**	−3.87	0.923	9.14 × 10^−5^	0.0242	Cell functions, cell type-specific, Th2 orientation
**PSEN1**	−1.47	0.361	0.000132	0.0316	T cell activation
**CASP3**	−1.46	0.365	0.000181	0.0401	Cell cycle checkpoint and arrest, co-regulators of autophagy and apoptosis/cell cycle, negative regulation of cell cycle
**DOCK9**	1.95	0.43	2.89 × 10^−5^	0.00995	Cell functions, cell type-specific
**TOLLIP**	2.33	0.498	1.74 × 10^−5^	0.00677	Innate immune response
**PNMA1**	2.46	0.623	0.000216	0.0447	Inflammatory response to antigenic stimulus
**MAPKAPK2**	2.5	0.495	4.45 × 10^−6^	0.00197	Innate immune response
**TFEB**	2.82	0.674	9.37 × 10^−5^	0.0242	Humoral immune response
**NT5E**	3.14	0.732	6.66 × 10^−5^	0.0207	CD molecules, phagocytosis
**IL1RAP**	4.25	0.774	8.96 × 10^−7^	0.000487	Innate immune response
**MNX1**	5.59	0.821	5.75 × 10^−9^	1.78 × 10^−5^	Humoral immune response
**TNFRSF11B**	7.24	1.22	1.55 × 10^−7^	0.000178	TNF superfamily members and their receptors
**NET G3/NEC**					
**MME**	−10	1.76	4.13 × 10^−7^	0.000427	Cell functions, cell type-specific
**ABCB1**	−5.49	0.925	1.66 × 10^−7^	0.000257	CD molecules (MDR)
**TPSAB1**	−5.11	1.27	0.00016	0.0496	Cell functions, cell type-specific
**MAF**	−4.32	0.882	7.95 × 10^−6^	0.00617	Cell functions, cell type-specific, Th2 orientation
**CASP1**	−4.2	0.691	9.57 × 10^−8^	0.000257	Innate immune response
**AMICA1**	−2.84	0.699	0.000146	0.0496	Regulation of immune response
**MIF**	2.43	0.52	1.80 × 10^−5^	0.0111	Innate immune response
**DUSP4**	4.61	1.01	2.70 × 10^−5^	0.014	Innate immune response
**TNFRSF11B**	4.73	1.16	0.000143	0.0496	TNF superfamily members and their receptors
**SPP1**	5.89	1.43	0.000123	0.0496	Cytokines and receptors

**Table 4:** Patient characteristics for samples used for digital spatial profiling.

	NET G3 (N)	NEC G3 (N)
**Patients**	18	11
**Stage II/III/IV/not known**	3/4/7/4	0/3/8/0
**Tissue samples**	20	13
**Primary tumor sites (N)**	Gastrointestinal (3), bile duct (1), pancreas (7), liver (1), prostate (1), ovary (1), skin (1), kidney (2), CUP (1)	Gastrointestinal (7), bile duct (2), (bladder (1), CUP (1)
Patients with primary tumor/metastasis analyzed *	18/2	11/1

* For three patients, primary tumor and metastasis sites were analyzed.

## Supplementary Materials

The following are available online at https://www.mdpi.com/2072-6694/12/11/3448/s1, Table S1: Detailed patients’ characteristics, Table S2: NanoString raw data, Table S3: DSP raw data.
